# The necessity for enhancing awareness of tuberculosis starting from the early college semesters: empirical evidence from a cross-sectional research

**DOI:** 10.3389/fpubh.2023.1272494

**Published:** 2023-10-26

**Authors:** Xinyi Chen, Ying Peng, Lin Zhou, Fei Wang, Bin Chen, Yan Qu

**Affiliations:** ^1^Department of Tuberculosis Control and Prevention, Zhejiang Provincial Centre for Disease Control and Prevention, Hangzhou, China; ^2^National Centre for Tuberculosis Control and Prevention, Chinese Center for Disease Control and Prevention, Beijing, China

**Keywords:** tuberculosis, awareness rate, health promotion and education, lower-grade students, questionnaire survey

## Abstract

**Background:**

College students, especially those in the lower grades, constitute the main high-risk population for tuberculosis (TB). Insufficient knowledge about TB among college students contributes to an increased risk of TB infection. In Zhejiang Province, China, limited research has been conducted recently on the awareness of TB in schools among college students. Therefore, this study aims to gain insight into TB knowledge among low-grade college students in Zhejiang Province and develop effective strategies for TB education targeted at this specific population.

**Methods:**

A cross-sectional survey was conducted between 1^st^ and 20^th^ May 2022 in 20 colleges in Zhejiang Province, southeastern China. The survey aimed to assess the level of TB awareness among 1^st^ and 2^nd^-year college students. Chi-square tests were performed to compare the rates, while multivariate logistic regression was used to identify the factors influencing the overall awareness level of students' regarding key knowledge about TB.

**Results:**

A total of 4,414 lower-grade students participated in the study. The total awareness rate and entire awareness rate of key TB knowledge were 81.6 and 25.3%, respectively. Participants who demonstrated a relatively poor understanding of the definition were (51.0%), curable outcomes (75.7%), and preventive measures of TB (76.1%). Female participants [adjusted odds ratio (aOR):1.44; 95% confidence interval (CI):1.25–1.65], medical students (aOR:2.00; 95%CI:1.63–2.64), had a high level of monthly expenditures (aOR:2.50; 95%CI:1.49–4.19), had prior TB health education (aOR:1.95; 95%CI:1.68–2.25) and previous exposure to TB patients (aOR:2.13; 95% CI:1.48–3.08) indicating a better level of awareness of key knowledge about TB. Among the students, 58.5% expressed their willingness to acquire TB knowledge through “broadcasting, television, films, and audiovisual materials.”

**Conclusions:**

The total awareness rate of key knowledge among low-grade college students in Zhejiang did not meet the national requirements. To effectively prevent TB in schools, it is crucial to develop a comprehensive understanding of the disease among college students. Therefore, it is necessary to enhance TB awareness through theoretical and practical education, starting from the early semesters of college.

## 1. Introduction

Tuberculosis (TB), an ancient disease caused by *Mycobacterium* tuberculosis (MTB), maintains its daunting position as the 13th leading cause of death worldwide and the leading cause of death from a single infectious agent since 2019 (1). Among the 22 high-burden countries, China ranks third and faces challenges in protecting high-risk, vulnerable, and special populations from TB (1). In 2018, nationwide TB cases reported among students reached 48,289, with an incidence rate of 17.97/100,000, representing a 29.19% increase compared to 2014 (13.91/100,000) (2). In many provinces of China, the number of students with TB ranks second, just behind farmers and herdsmen (3).

In recent years, the number of college students in China has been increasing due to the dramatic expansion of college enrolment (4). Hence, TB prevention and control among colleges have become increasingly important (5). Previous studies have identified college and university students aged 19–22 years as the main population at high-risk for TB (6). Factors such as high population density, close contact, and increased mobility contribute to TB infection and transmission among college students. Additionally, a lack of awareness about TB is considered an essential factor that increases the risk of exposure to the disease (7). However, a previous survey indicated inadequate knowledge of TB among students (8). Several studies have shown that even when many 1^st^-year students exhibited suspicious symptoms of TB, such as coughing and sputum for more than 2 weeks (9–11), both patients themselves and their fellow classmates lacked awareness of TB. This led to delays in seeking medical care and reporting to the school medical staff, resulting in a high prevalence of TB in the colleges (12).

Zhejiang Province in southeastern China serves as a representative province, which is composed of 11 prefectures and 90 county-level administrative regions. It is one of the most densely populated provinces in China, with the fourth highest gross domestic product (GDP) and a continuously growing migrant population. According to the report of the fourth provincial TB key awareness investigation, the total awareness rate of the public in Zhejiang Province was 48.0%, of which only 38.0% was among students, the lowest among all walks of life (13). In 2021, the Tuberculosis Information Management System (TBIMS) reported more than 30 school-related TB outbreaks in the province, with approximately one-third of these outbreaks occurring in colleges. College students come from diverse regions across the country, and their living environments and study patterns have changed significantly compared to those of high school students. In particular, junior students who have recently entered college may struggle to adapt to a new environment and may become prone to TB once their resistance decreases. A study showed that the prevalence rates of active TB among college students in China ranged from 0.40 to 1.52%, with outbreak rates as high as 2.89–4.20%, significantly higher than the rate in the general population (14). Currently, TB prevention in colleges mainly relies on TB health education, which is widely considered an effective approach to promote TB knowledge among students. College students, being a population with a high incidence of TB possess a higher level of knowledge and acceptance and a stronger sense of social responsibility. Therefore, health education on TB is easily implementable and accepted among them, yielding efficient results in the prevention of TB. However, there have been limited studies specifically investigating the level of TB awareness among low-grade college students in recent years. This lack of research impedes the scientific evaluation of the effectiveness of current TB education methods. Hence, this study targeted low-grade college students to assess their level of TB awareness and explore the sociodemographic determinants associated with their perception of this infectious disease. The findings of this study will provide a basis for optimizing TB prevention and control strategies in colleges and universities.

## 2. Materials and methods

### 2.1. Study setting

A cross-sectional survey was conducted between 1^st^ and 20^th^ May 2022 in 20 colleges in Zhejiang Province, southeastern China, and focused on the assessment of key TB knowledge among low-grade college students. As of 2022, Zhejiang Province was home to 109 institutions of higher education, mainly distributed in the southern and northern regions, ranking eleventh among all Chinese provinces.

### 2.2. Participants

A cluster random sampling strategy was used for participants selection. Specifically, 20 colleges were randomly selected from four cities within the north and south of the Zhejiang Province: Hangzhou (eight), Ningbo (five), Wenzhou (five), and Jiaxing (two). Approximately 8 to 10 freshman and sophomore classes were randomly selected from the selected samples, and 20 to 25 male and female students in these classes were randomly selected for the study. Totally 4,414 subjects were investigated in this study.

### 2.3. Questionnaire and quality control

In this study, a unified electronic questionnaire was adopted to collect information, which was formulated based on the Technical specifications for tuberculosis prevention and control in China (2020 Edition) (15) and the School TB Prevention and Control Work Specification (2017 Edition) (16), as well as literature and expert consultations, including three aspects: participants' sociodemographic characteristics, knowledge of key information regarding TB occurrence in schools, and accessibility to TB knowledge. The key knowledge of TB included eight items (see Supplementary Table S1) with several options for TB key knowledge, which contained only one correct answer for each item.

The local Centre for Disease Control and Prevention (CDC) staff at each investigated school were responsible for the interviews and collection of the questionnaires. These investigators received provincial training and followed unified investigation guidelines to ensure authenticity and integrity of the entire process. The provincial CDC routinely verified the responses provided in the electronic questionnaire daily, including checking the number, completeness, and logical coherence of the responses, facilitating the identification and timely rectification of any errors. The final questionnaire database was uniformly established at the provincial level, and quality verification was performed.

### 2.4. Definition of items

In this study, low-grade students included 1^st^- and 2^nd^-year undergraduate students at these 20 colleges. The total awareness rate of the eight key TB information items was calculated by dividing the number of correct answers provided by all participants by the total number of answers to the eight questions. The entire awareness rate represents the percentage of participants who correctly answered all key TB knowledge questions out of the total number of survey participants. The awareness rate of each key TB knowledge item was calculated as the percentage of participants who correctly answered the questions for that item out of the total number of participants who answered it. The average of the correct answers given by all participants was used to divide the awareness level, with scores higher than the average indicating good awareness and scores lower than the average indicating poor awareness.

### 2.5. Statistic analysis

The survey data were collated using Microsoft Excel and exported to SPSS package, v24.0.0 (IBM Corporation, Armonk, NY, USA), for analysis. Descriptive data were summarized and presented as frequencies and percentages. A chi-square test was performed to assess the awareness levels of participants with different characteristics. Covariates with *p-*values < 0.10 in the univariable analysis were considered for inclusion in the multivariable logistic regression model. Adjusted odds ratios (aOR) and 95% confidence intervals (CI) were presented to show the association between participant characteristics and their awareness level of TB. A *p*-value ≤ 0.05 was considered statistically significant.

## 3. Results

### 3.1. Demographic characteristics of participants

**Figure 1:**
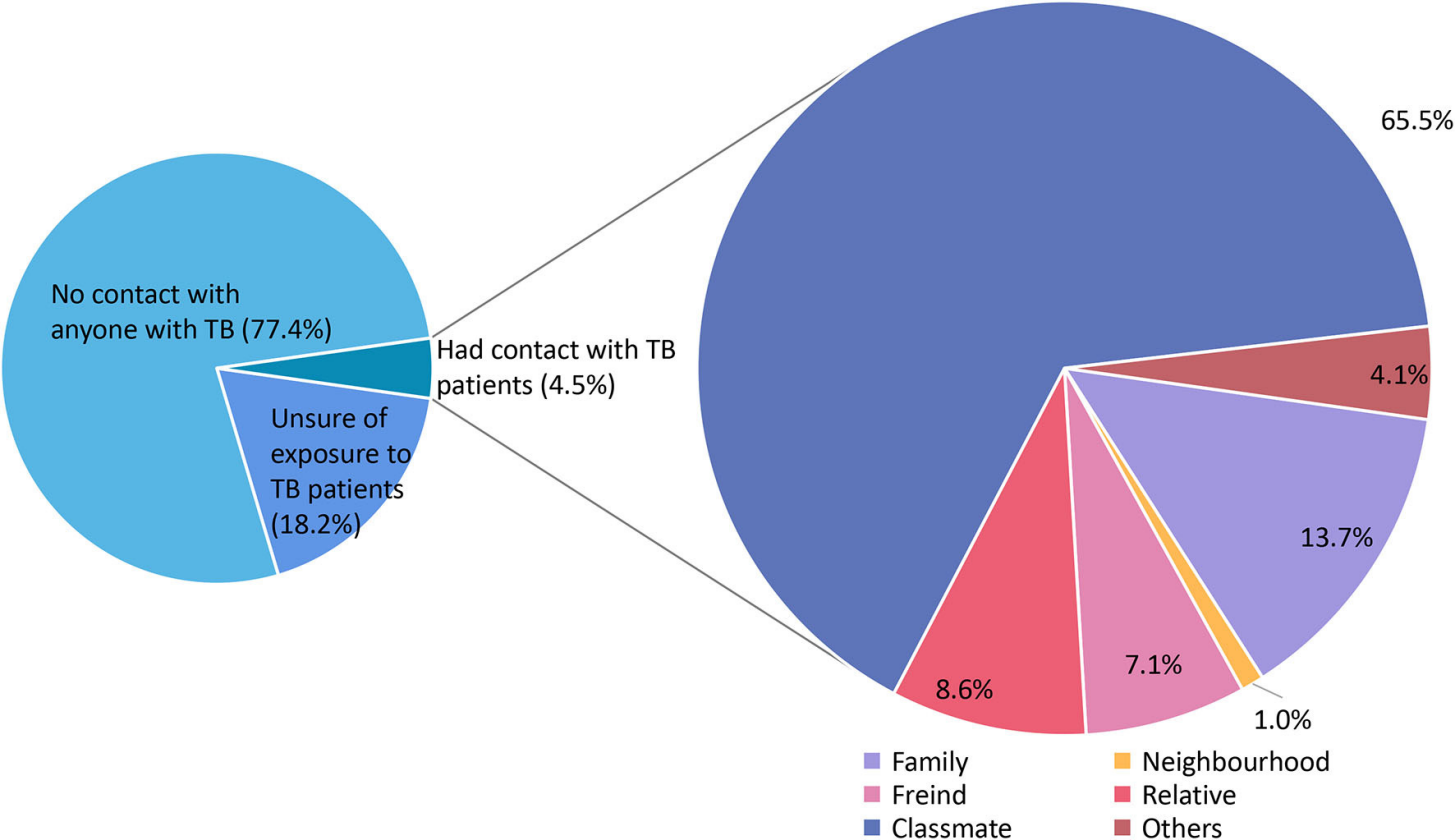
Categories of TB patients that the participants had ever contacted.

As presented in Table 1, a total of 4,414 questionnaires were collected. The majority of participants were 20 years old (42.8%). The proportion of females was slightly higher than that of males (51.7 vs. 48.3%, respectively). The Han ethnic group constituted the vast majority of the population (96.1%). Nearly 64.6% of participants resided in rural areas. Approximately 71.5% of the participants attended high school in Zhejiang Province. Among all the low-grade college students, freshmen accounted for 53.9%. Science and engineering professions had the highest group of participants (45.1%), followed by humanities and social sciences (31.0%). The majority of participants' parents were junior high school graduates, accounting for 40.0%. Nearly 70.3% of the participants had a monthly spending range between 1,000 and 2,000 RMB. A total of 77.1% of participants indicated that they had received previous knowledge about TB. Only 0.6% of the participants had suffered from TB, and 4.5% had previous exposure to patients with TB, while the 77.4% of the participants had not been exposed to patients with TB, and 18.2% responded that they did not know. Figure 1 further demonstrated the relationship between participants who responded that they had been exposed to a TB patient and the patient. Among 197 participants who had been exposed to TB patients, approximately 65.5% of the TB patients were their classmates, followed by family members (13.7%).

**Table 1:** Demographic characteristics of participants (*n* = 4,414).

**Demographic factors**	**Number**	**Percentage (%)**
**Age**		
< 20	1,498	33.9
20	1,890	42.8
>20	1,026	23.2
**Sex**		
Male	2,282	51.7
Female	2,132	48.3
**Nationality**		
Han	4,244	96.1
Others	170	3.9
**Area residence**		
Urban	1,562	35.4
Rural	2,852	64.6
**Origin of the students**		
Zhejiang Province	3,157	71.5
Others	1257	28.5
**Grade**		
Year 1	2,378	53.9
Year 2	2,036	46.1
**Discipline of study**		
Humanity social sciences	1,367	31.0
Science and engineering	1,989	45.1
Medicine	675	15.3
Art	383	8.7
**Father's level of education**		
Illiterate	46	1.0
Primary school	706	16.0
Middle school	1,766	40.0
High school	935	21.2
University/College	890	20.2
Postgraduate	71	1.6
**Mother's level of education**		
Illiterate	97	2.2
Primary school	952	21.6
Middle school	1,706	38.6
High school	832	18.8
University/College	772	17.5
Postgraduate	55	1.2
**Monthly living expenses (RMB)**		
≤ 500	72	1.6
501–1,000	318	7.2
1,001–1,500	1,353	30.7
1,501–2,000	1,752	39.7
≥2,000	919	20.8
**Have you previously received information about TB?**		
Yes	3,404	77.1
No	1,010	22.9
**Have you ever had TB?**		
Yes	4,387	99.4
No	27	0.6
**Have you ever had contact with a TB patient?**		
Yes	197	4.5
No	3,415	77.4
No idea	802	18.2

### 3.2. Awareness of TB key knowledge

**Figure 2:**
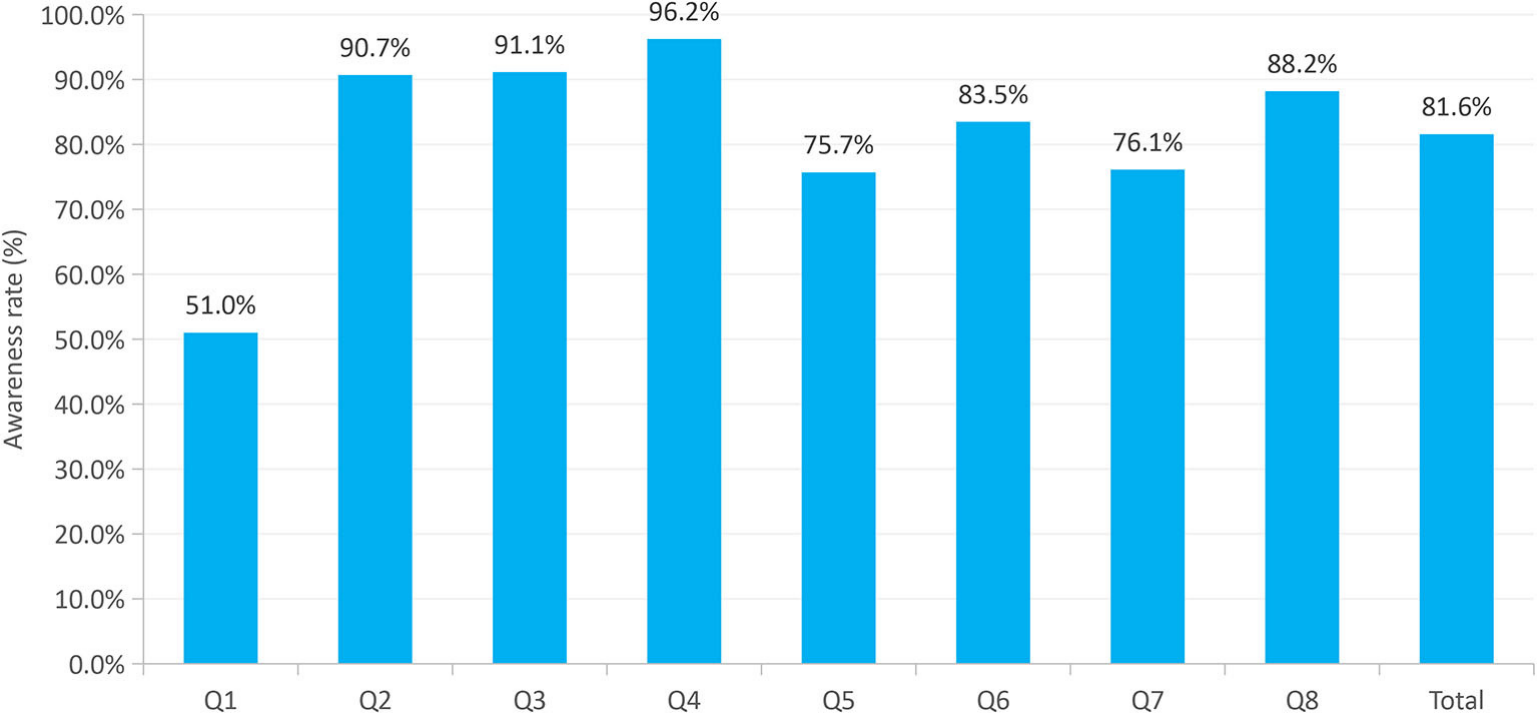
Participants' awareness rate of each TB key knowledge among low-grade college students in Zhejiang Province, southeastern China. Q1: what is TB; Q2: how is TB transmitted; Q3: what are the suspicious symptoms of TB; Q4: if you suspect that you have TB, what should you do; Q5: is TB curable; Q6: while at school, when you develop suspicious symptoms of TB or are diagnosed with TB, what should you do; Q7: what practice is beneficial in preventing the spread of TB; Q8: which lifestyle habit will improve your immunity?

Overall, the participants' total awareness rate for the eight key knowledge items in the survey was 81.6% (Figure 2). For each of the key knowledge, the participants' awareness level of TB as a chronic infectious disease was the lowest (Q1), at 51.0%, followed by the curability of TB with a correct response of 75.7% (Q5). However, the participants demonstrated good knowledge of the TB transmission modes (Q2, 90.0%), suspicious symptoms (Q3, 91.1%), and where to seek medical help after suspected TB (Q4, 96.2%), with awareness rates for all three types of information exceeding 90.0%. The awareness of each key knowledge of TB among the different characteristics of the participants is shown in Supplementary Table S2.

Table 2 presents the details of the responses regarding key TB knowledge. In this study, approximately half of the participants (51.0%) were aware that TB is a chronic communicable disease, while 31.5% recognized it as a communicable disease. The majority (90.7%) correctly stated that TB is transmitted through the respiratory tract. Approximately 91.1 and 96.2% of participants correctly knew the suspected symptoms of TB and where to seek medical help, respectively. Regarding curability of TB, 13.8% of participants responded that they did not know, while 4.3% believed it was not curable. When asked about students being diagnosed with TB at school, 14.9% believed immediate hospitalization was necessary, and 83.5% of the participants agreed that they should be proactive in reporting their illness to school rather than hiding it or bringing it to class. Most participants (76.1%) mentioned appropriate methods for preventing TB transmission. However, about 3.2% of participants described inappropriate lifestyle habits, including staying up late, overeating, occasional exercise, and irregular exercise or diet.

**Table 2:** Responses to eight TB key knowledge among low-grade college students in Zhejiang Province, southeastern China.

**Questions**	**Number**	**Percentage (%)**
**Q1: What is TB?**		
Acute communicable disease	1,390	31.5
Acute non-communicable diseases	345	7.8
Chronic communicable diseases	2,251	51.0
Chronic non-communicable diseases	428	9.7
**Q2: How is TB transmitted?**		
No idea	272	6.2
Through respiratory tract transmission	4,003	90.7
Through digestive tract transmission	78	1.8
Through blood-borne transmission	61	1.4
**Q3: What are the suspicious symptoms of TB?**		
No idea	235	5.3
Abdominal pain and diarrhea	25	0.6
Cough and sneeze	131	3.0
Cough, sputum for more than 2 weeks or sputum with blood	4,023	91.1
**Q4: If you suspect that you have TB, what should you do?**		
No idea	131	3.0
Visit local TB designated medical institutions	4,248	96.2
Visit a private clinic	18	0.4
Buy your own medicine	17	0.4
**Q5: Is TB curable?**		
No idea	611	13.8
Most of them can be cured	3,341	75.7
All of them can be cured	273	6.2
Incurable	189	4.3
**Q6: While at school, when you develop suspicious**		
**symptoms of TB or are diagnosed with TB, what should you do?**		
Take lessons while in therapy	27	0.6
Immediate hospitalization	659	14.9
Observation without treatment if symptoms are not severe	43	1.0
Take the initiative to report to the school and do not conceal your illness or bring it to class	3,685	83.5
**Q7: What practice is beneficial in preventing the spread of TB?**		
Not drinking raw water	392	8.9
No littering	100	2.3
Regular diet	562	12.7
Open windows regularly for ventilation	3,360	76.1
**Q8: Which lifestyle habit will improve your immunity?**		
Stay up late, overeat and exercise occasionally	44	1.0
Stay up late, irregular diet, lack of exercise	98	2.2
Wear a mask, eat a balanced diet and exercise consistently	379	8.6
Keep early hours, balanced diet and consistent exercise	3,893	88.2

**Figure 3:**
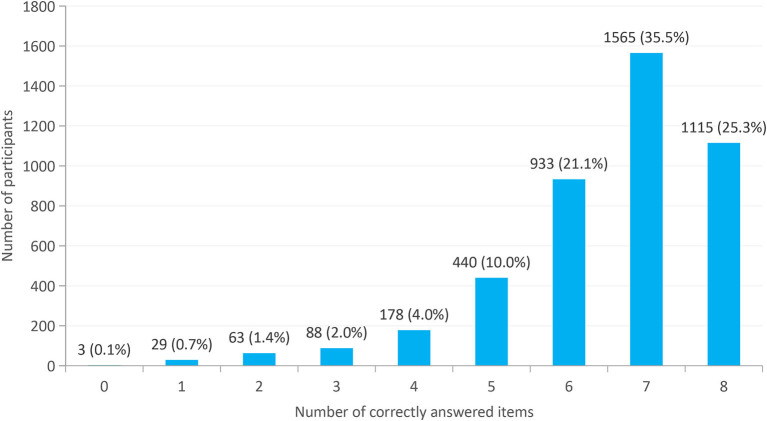
Percentage of participants who correctly answered the number of TB key knowledge items.

The average of correct answers given by all participants was approximately 6.5. Therefore, participants who correctly answered seven and eight key knowledge items were classified as having good awareness, and participants who correctly answered less than or equal to six key knowledge items were classified as having poor awareness. As shown in Figure 3, the total number of students who correctly answered more than six messages was 2,680 (accounting for 60.7%). Three participants (0.1%) incorrectly answered all eight core messages, and the entire awareness rate was only 25.3%.

### 3.3. Factors associated with the level of awareness

As presented in Table 3, the level of awareness regarding key TB knowledge among lower-grade college students varied by sex (*p* < 0.001), place of residence (*p* = 0.013), discipline of study (*p* < 0.001), monthly living expenses (*p* < 0.001), prior knowledge about TB (*p* < 0.001), and exposure to TB patients (*p* < 0.001). A greater proportion of females (65.5%) had higher awareness levels than males (56.3%). Approximately 63.2% of participants residing in urban areas had a good level of awareness. Among different disciplines, students in the field of medicine had the highest proportion with good awareness of TB, accounting for 73.3%. The proportion of participants with better awareness of TB tended to increase with higher monthly living expenses. Students who had received prior TB education (64.4%) and those who had been in contact with TB patients (76.6%) had a higher percentage of good awareness level.

**Table 3:** Comparison of the awareness level of TB among low-grade college students with different characteristics in Zhejiang Province, southeastern China.

**Demographic factors**	**Awareness level**		***p*-value**
	**Good**, ***n*** **(%)**	**Poor**, ***n*** **(%)**	
**Age of year**			0.773
< 20	919 (61.3)	579 (38.7)	
20	1,146 (60.6)	744 (39.4)	
>20	615 (59.9)	411 (40.1)	
**Sex**			< 0.001
Male	1,284 (56.3)	998 (43.7)	
Female	1,396 (65.5)	736 (34.5)	
**Nationality**			0.248
Han	2,584 (60.9)	1,660 (39.1)	
Others	96 (56.5)	74 (43.5)	
**Area residence**			0.013
Urban	987 (63.2)	575 (36.8)	
Rural	1,693 (59.4)	1,159 (40.6)	
**Origin of the students**			0.063
Zhejiang Province	1,944 (61.6)	1,213 (38.4)	
Others	736 (58.6)	521 (41.4)	
**Grade**			0.673
Year 1	1,437 (60.4)	941 (39.6)	
Year 2	1,243 (61.1)	793 (38.9)	
**Discipline of study**			< 0.001
Humanity social sciences	815 (59.6)	552 (40.4)	
Science and engineering	1,139 (57.3)	850 (42.7)	
Medicine	495 (73.3)	180 (26.7)	
Art	231 (60.3)	152 (39.7)	
**Monthly living expenses (RMB)**			< 0.001
≤ 500	24 (33.3)	48 (66.7)	
501–1,000	184 (57.9)	134 (42.1)	
1,001–1,500	834 (61.6)	519 (38.4)	
1,501–2,000	1,082 (61.8)	670 (38.2)	
≥2,000	556 (60.5)	363 (39.5)	
**Have you previously received information about TB?**			< 0.001
Yes	2,193 (64.4)	1,211 (35.6)	
No	487 (48.2)	523 (51.8)	
**Have you ever had TB?**			0.058
Yes	17 (63.0)	10 (37.0)	
No	2,663 (60.7)	1,724 (39.3)	
**Have you ever had contact with a TB patient?**			< 0.001
Yes	151 (76.6)	46 (23.4)	
No	2,062 (60.4)	1,353 (39.6)	
No idea	467 (58.2)	335 (41.8)	
Total	2,680 (60.7)	1,734 (39.3)	

Table 4 demonstrates the results of multivariate logistic regression model, indicating that females were more likely to have a better awareness of key TB knowledge compared to males (aOR: 1.44; 95%CI: 1.25–1.65). Medical students exhibited significantly higher levels of TB awareness compared to students in humanities and social sciences (aOR: 2.00; 95%CI: 1.63–2.64). Additionally, students with higher living costs were more knowledgeable than those with lower living costs. There was also a positive association between receiving knowledge about TB and better awareness (aOR: 1.95; 95%CI: 1.68–2.25). Participants previously exposed to TB patients were approximately twice as likely to have higher levels of TB key knowledge than those who were unsure if they had been exposed (aOR: 2.13; 95%CI: 1.48–3.08).

**Table 4:** Factors associated with the level of awareness toward TB among low-grade college students with different characteristics in Zhejiang Province, southeastern China.

**Variables^a^**	**aOR^b^**	**95%CI**	***p*-value**
**Sex**			
Male	1		
Female	1.44	1.25–1.65	< 0.001
**Discipline of study**			
Humanity social sciences	1		
Science and engineering	1.08	0.92–1.26	0.361
Medicine	2.00	1.63–2.46	< 0.001
Art	1.09	0.86–1.38	0.487
**Monthly living expenses (RMB)**			
≤ 500	1		
501–1,000	2.66	1.54–4.59	< 0.001
1,001–1,500	2.90	1.74–4.83	< 0.001
1,501–2,000	2.66	1.60–4.43	< 0.001
≥2,000	2.50	1.49–4.19	< 0.001
**Have you previously received information about TB?**			
No	1		
Yes	1.95	1.68–2.25	< 0.001
**Have you ever had contact with a TB patient?**			
No idea	1		
Yes	2.13	1.48–3.08	< 0.001
No	1.05	0.90–1.24	0.510

^a^Variables: variables of age of year, nationality, area residence, origin of the students, grade and whether had TB before were excluded by the logistic stepwise regression. ^b^aOR: adjusted odds ratio.

### 3.4. The preference for the ways of TB health education

**Figure 4:**
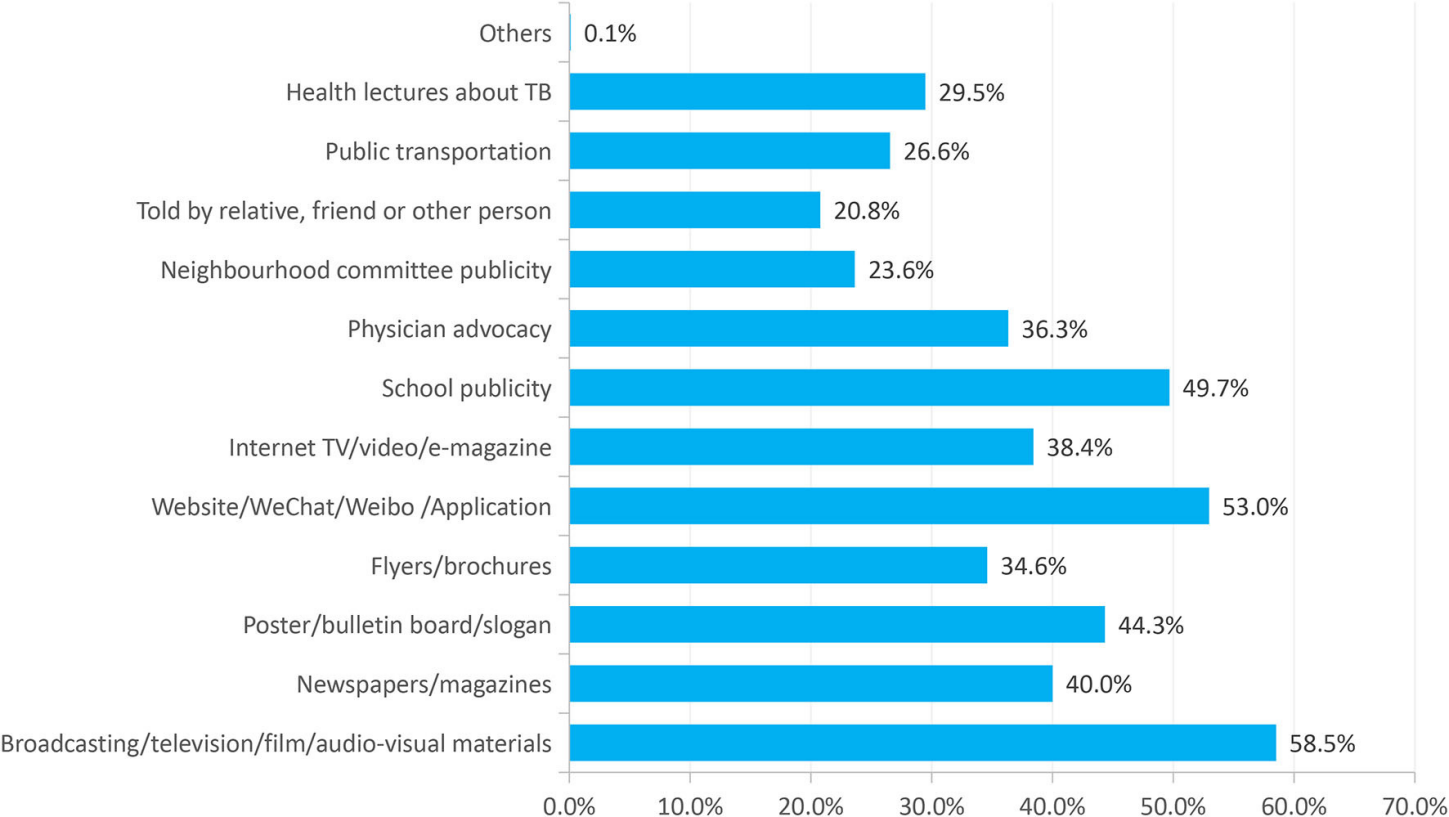
Preferences of low-grade college students for the ways to receive TB knowledge.

In Figure 4, regarding the ways of acquiring knowledge about TB, 58.5, 53.0, and 49.7% of the students expressed willingness to receive TB knowledge through “Broadcasting/television/film/audio-visual materials,” “Website/WeChat/Weibo/Application,” and “School publicity,” respectively.

## 4. Discussion

Students constitute a pivotal demographic in the prevention and control of TB, and their comprehension of this disease is indispensable in curbing its incidence among them. In this study, a deeper understanding of the actual circumstances faced by lower-grade college students regarding TB knowledge was obtained through a validated questionnaire. The overall awareness rate of key TB knowledge among low-grade college students in Zhejiang Province in 2022 was 81.6%, which was lower compared to 89.02% in Jiangsu Province (17) but higher than 73.3% in Guangxi Zhuang Autonomous Region (4). Nevertheless, a gap remains to be bridged to achieve the target (≥85%) among populations proposed by the “13^th^ Five-Year” National TB Control Plan in China. This indicates that educational interventions are still necessary to improve TB knowledge among college students.

Assessing students' knowledge of TB based solely on total knowledge is flawed as the overall knowledge rate decreases with increasing key knowledge questions (17). Therefore, It should be evaluated in conjunction with the entire awareness rate. In our study, the overall awareness rate among all low-grade college students was only 25.3%, which was lower than the score (43.45%) reported in a previous study conducted in Hunan Province (18), but higher than the 16.03% reported in a survey conducted in Beijing (19). A survey assessing students' knowledge of four infectious diseases (Hepatitis B, Hepatitis C, AIDS, and TB) revealed a high error rate in the awareness of these diseases, except for AIDS (20), which was consistent with the results of our study.

Our survey revealed an imbalanced in the awareness levels of eight key aspects of TB knowledge among lower-grade college students, with awareness rates ranging from 51.0 to 96.2%. This indicates that the students' understanding of TB prevention and control is not comprehensive, necessitating the implementation of various forms of education to address these knowledge gaps. Specifically, students demonstrated confusion regarding the definition of TB, particularly in distinguishing whether it is an acute or a chronic infectious disease. Consequently, this key message had the lowest awareness rate among all items of TB core knowledge. Some students expressed uncertainty about the curability of TB, while others were unsure about the preventive measures against TB transmission. Despite these gaps, lower-grade students exhibited a high level of awareness about TB transmission routes, suspicious symptoms, the importance of not concealing physical conditions, seeking immediate treatment from designated medical institutions for TB consultation upon illness, and adopting an immune-enhancing lifestyle. These findings were consistent with previous research (4, 8, 21), indicating a positive trend in health-seeking behavior among our students. Notably, several studies have established an association between TB perceptions and early treatment-seeking (22, 23).

Regarding the factors influencing TB awareness level, it was found that the discipline of study was one of the crucial factors affecting the awareness level toward TB knowledge among low-grade college students. Medical students had significantly higher awareness levels than students in other disciplines, similar to the findings of surveys conducted at 15 Italian and Jordanian universities (24, 25). This may be related to the possibility that medical students are exposed to more medical knowledge and exhibit more interest in medical-related content during their study practice. However, medical students' awareness of the definition (57.2%) and curability (81.2%) of TB was relatively weak. This might be because 1^st^-year and 2^nd^-year medical students have just started to come into contact with clinical professional knowledge, and their mastery of the specialty is not yet comprehensive. However, medical students in universities or colleges can be exposed to TB infections during clinical rotations (26), and an inadequate knowledge of TB can increase their risk of infection. As potential future physicians or leaders in the fight against TB, medical students' knowledge toward TB will directly impact future efforts aimed at controlling the disease. Therefore, raising awareness of TB among medical students should be considered an important issue in medical education. Besides, in this study, lower-grade students with a higher overall awareness rate of the eight core knowledge of TB were those who had suffered TB or received TB health education before enrolment, which is consistent with similar studies findings (27–29). TB health education is a crucial strategy in China's effort to control TB, aiming to raise awareness of TB control policies and knowledge among diverse groups, enabling them to adopt appropriate behaviors and implement effective TB control strategies. By doing so, the ultimate goal of bringing the TB epidemic under control can be achieved. Although, there has been continuous focus on TB health education and promotion for the general public, particular attention has been given to TB patients and students in China (30). However, the state of TB health education and promotion in universities and colleges remains sub-optimal. This current scenario indicates the urgent need to strengthen theoretical and practical health education on TB from the beginning of university or college enrolment. Since students are accustomed to receiving education while in school, they are more receptive and responsive to targeted health education messages and are more inclined to absorb the information and disseminate TB knowledge to their families and communities through the “teacher-student-parent-community” chain (4, 31). This, in turn, has a significant impact on raising TB awareness throughout society.

College students are currently in the midst of a digital age, where mobile phones and computers have become the primary means of socializing and acquiring knowledge (32). Our study revealed that students were more likely to receive TB health education through news media and the Internet, compared to traditional promotional materials. Therefore, taking advantage of the above-mentioned means and methods of publicity can be effectively implemented. In addition, focus should be placed on the development and use of news media platforms such as the Internet, Weibo, and WeChat- to promote TB education, cultivate more student volunteers to promote TB prevention, and facilitate peer education. The rapid development of the Internet has broadened students' access to the world; however, the quality of information in the cyber world cannot be guaranteed, and students are susceptible to receiving inaccurate information. Thus, agencies involved in disease control and prevention should increase online publicity and strengthen the supervision of false disease information on the Internet to ensure students obtain accurate knowledge on disease prevention.

This study employed an innovative approach in the Internet Era by utilizing an electronic questionnaire, which provides new ideas and methods for disseminating knowledge and investigating TB prevention and control in schools. However, this study has some limitations. First, due to geographical constraints, economic conditions, and low prevalence of TB in Zhejiang Province, our findings may not fully represent TB awareness among lower-grade college students nationwide, especially in economically disadvantaged and high-prevalence areas of the disease. Second, since this study involved 20 colleges, we had to use an open online questionnaire platform and did our best to ensure that the survey reached only the target population. Nevertheless, we acknowledge the possibility of non-students receiving the survey link and completing the questionnaire. Third, as this was a cross-sectional study, we could not draw a causal relationship between the factors and their effects.

## 5. Conclusion

The total awareness rate of key TB knowledge among college students in Zhejiang Province fell short of national requirements, indicating a generally low level of awareness. Schools face significant and arduous tasks in TB prevention and control. To improve TB awareness among students, it is necessary to constantly innovate the content and delivery of health education and carry out comprehensive and multi-dimensional publicity activities suitable for school health education and promotion, with particular attention given to addressing the weak points in students' TB knowledge. Targeted health education on key TB knowledge should be provided, taking into account the preferences and needs of students at different levels and grades. This approach will contribute to fostering improved behavioral habits and strengthening the ability to prevent and control TB.

## Data availability statement

The original contributions presented in the study are included in the article/Supplementary material, further inquiries can be directed to the corresponding authors.

## Ethics statement

The studies involving human participants were reviewed and approved by Zhejiang Provincial Centre for Disease Control and Prevention. The participants provided their written informed consent to participate in this study.

## Author contributions

XC: Formal analysis, Funding acquisition, Methodology, Writing—original draft, Writing—review & editing. YP: Formal analysis, Methodology, Writing—review and editing. LZ: Formal analysis, Writing—review & editing. FW: Formal analysis, Writing—review & editing. BC: Conceptualization, Funding acquisition, Project administration, Supervision, Writing—review & editing. YQ: Conceptualization, Project administration, Supervision, Writing—review & editing.
