# Supplementary material for: The necessity for enhancing awareness of tuberculosis starting from the early college semesters: empirical evidence from a cross-sectional research
Source: Front Public Health. 2023 Oct 26;11:1272494. doi: 10.3389/fpubh.2023.1272494 (PMC10637372; doi:10.3389/fpubh.2023.1272494)
Supplement: Supplementary file 1 [file Table_1.DOCX]

**Table S1** Eight key knowledge of TB prevention and control in school

| **Question** | **Key Knowledge** |
| --- | --- |
| Q1: What is TB? | TB is a chronic communicable disease that seriously harms people's health for a long time. |
| Q2: How is TB transmitted? | TB spreads mainly through the respiratory tract and everyone can be infected. |
| Q3: What are the suspicious symptoms of TB? | TB should be suspected if a cough or expectoration persists for more than two weeks and should be treated in designated TB hospitals immediately. |
| Q4: If you suspect that you have TB, what should you do? | Go to local TB designated medical institutions for testing, treatment and management. |
| Q5: Is TB curable? | Standardize the whole-course treatment, the vast majority of TB patients can be cured, but also to avoid infecting others. |
| Q6: While at school, when you develop suspicious symptoms of TB or are diagnosed with TB, what should you do? | After showing suspicious symptoms of TB or being diagnosed as a TB patient, the student should take the initiative to report to the school, do not conceal his/her disease condition, and do not attend classes with illness. |
| Q7: What practice is beneficial in preventing the spread of TB? | Develop the habit of frequently opening windows for ventilation. Do not spit everywhere, cover your mouth and nose when coughing and sneezing, and wear a mask to reduce the spread of TB. |
| Q8: Which lifestyle habit will improve your immunity? | Ensure ample sleep, reasonable diet, strengthen physical exercise, build up resistance to disease. |
