# Supplementary material for: The necessity for enhancing awareness of tuberculosis starting from the early college semesters: empirical evidence from a cross-sectional research
Source: Front Public Health. 2023 Oct 26;11:1272494. doi: 10.3389/fpubh.2023.1272494 (PMC10637372; doi:10.3389/fpubh.2023.1272494)
Supplement: Supplementary file 2 [file Table_2.DOCX]

**Table S2** Awareness rates of TB key knowledge among junior college students with different characteristics in Zhejiang Province, southeast China

| **Demographic factors** | **Q1, n (%)** | **Q2, n (%)** | **Q3, n (%)** | **Q4, n (%)** | **Q5, n (%)** | **Q6, n (%)** | **Q7, n (%)** | **Q8, n (%)** |
| --- | --- | --- | --- | --- | --- | --- | --- | --- |
| **Age of year** |  |  |  |  |  |  |  |  |
| <20 | 760 (50.7) | 1376 (91.9) | 1364 (91.1) | 1443 (96.33) | 1138 (75.97) | 1256 (83.85) | 1126 (75.17) | 1335 (89.12) |
| 20 | 991 (52.4) | 1716 (90.8) | 1730 (91.5) | 1823 (96.46) | 1426 (75.45) | 1575 (83.33) | 1441 (76.24) | 1655 (87.57) |
| >20 | 500 (48.7) | 911 (88.8) | 929 (90.55) | 982 (95.71) | 777 (75.73) | 854 (83.24) | 793 (77.29) | 903 (88.01) |
| **Sex** |  |  |  |  |  |  |  |  |
| Male | 1116 (48.9) | 2038 (89.3) | 2017 (88.4) | 2151 (94.3) | 1653 (72.4) | 1834 (80.4) | 1738 (76.2) | 1953 (85.6) |
| Female | 1135 (53.2) | 1965 (92.2) | 2006 (94.1) | 2097 (98.4) | 1688 (79.2) | 1851 (86.8) | 1622 (76.1) | 1940 (91.0) |
| **Nationality** |  |  |  |  |  |  |  |  |
| Han | 2162 (50.9) | 3848 (90.7) | 3876 (91.3) | 4084 (96.2) | 3214 (75.7) | 3545 (83.5) | 3230 (76.1) | 3749 (88.3) |
| Others | 89 (52.4) | 155 (91.2) | 147 (86.5) | 164 (96.5) | 127 (74.7) | 140 (82.4) | 130 (76.5) | 144 (84.7) |
| **Area residence** |  |  |  |  |  |  |  |  |
| Urban | 853 (54.6) | 1424 (91.2) | 1442 (92.3) | 1506 (96.4) | 1206 (77.2) | 1319 (84.4) | 1227 (78.6) | 1371 (87.8) |
| Rural | 1398 (49.0) | 2579 (90.4) | 2581 (90.5) | 2742 (96.1) | 2135 (74.9) | 2366 (83.0) | 2133 (74.8) | 2522 (88.4) |
| **Origin of the students** |  |  |  |  |  |  |  |  |
| Zhejiang Province | 1578 (50.0) | 2883 (91.3) | 2883 (91.3) | 3030 (96.0) | 2417 (76.6) | 2645 (83.8) | 2418 (76.6) | 2782 (88.1) |
| Others | 673 (53.5) | 1120 (89.1) | 1140 (90.7) | 1218 (96.9) | 924 (73.5) | 1040 (82.7) | 942 (74.9) | 1111 (88.4) |
| **Grade** |  |  |  |  |  |  |  |  |
| Year 1 | 1173 (49.3) | 2172 (91.3) | 2164 (91.0) | 2295 (96.5) | 1810 (76.1) | 1998 (84.0) | 1800 (75.7) | 2110 (88.7) |
| Year 2 | 1078 (52.9) | 1831 (89.9) | 1859 (91.3) | 1953 (95.9) | 1531 (75.2) | 1687 (82.9) | 1560 (76.6) | 1783 (87.6) |
| **Major** |  |  |  |  |  |  |  |  |
| Humanity Social Sciences | 691 (50.5) | 1230 (90.0) | 1246 (91.1) | 1322 (96.7) | 1038 (75.9) | 1129 (82.6) | 1028 (75.2) | 1201 (87.9) |
| Science and Engineering | 979 (49.2) | 1786 (89.8) | 1784 (89.4) | 1898 (95.4) | 1467 (73.8) | 1619 (81.4) | 1481 (74.5) | 1736 (87.3) |
| Medicine | 386 (57.2) | 638 (94.5) | 637 (94.4) | 658 (97.5) | 548 (81.2) | 604 (89.5) | 558 (82.7) | 611 (90.5) |
| Art | 195 (50.9) | 349 (91.1) | 356 (93.0) | 370 (96.6) | 288 (75.2) | 333 (86.9) | 293 (76.5) | 345 (90.1) |
| **Monthly living expenses (RMB)** |  |  |  |  |  |  |  |  |
| ≤500 | 30 (41.7) | 58 (80.6) | 56 (77.8) | 62 (86.1) | 44 (61.1) | 50 (69.4) | 47 (65.3) | 54 (75.0) |
| 501-1000 | 162 (50.9) | 283 (89.0) | 284 (89.3) | 301 (94.7) | 231 (72.6) | 264 (83.0) | 241 (75.8) | 274 (86.2) |
| 1001-1500 | 688 (50.8) | 1221 (90.2) | 1244 (91.9) | 1315 (97.2) | 1033 (76.3) | 1131 (83.6) | 1042 (77.0) | 1205 (89.1) |
| 1501-2000 | 904 (51.6) | 1623 (92.6) | 1614 (92.1) | 1703 (97.2) | 1341 (76.5) | 1474 (84.1) | 1347 (76.9) | 1560 (89.0) |
| ≥2000 | 467 (50.8) | 818 (89.0) | 825 (89.8) | 867 (94.3) | 692 (75.3) | 766 (83.4) | 683 (74.3) | 800 (87.1) |
| **Have you previously received information about TB?** |  |  |  |  |  |  |  |  |
| Yes | 7166 (51.9) | 3183 (93.5) | 3158 (92.8) | 3317 (97.4) | 2706 (79.5) | 2843 (83.5) | 2653 (77.9) | 3009 (88.4) |
| No | 485 (48.0) | 820 (81.2) | 865 (85.6) | 931 (92.2) | 635 (62.9) | 842 (83.4) | 707 (70.0) | 884 (87.5) |
| **Have you ever had TB?** |  |  |  |  |  |  |  |  |
| Yes | 15 (55.6) | 24 (88.9) | 24 (88.9) | 25 (92.6) | 19 (70.4) | 22 (81.5) | 22 (81.5) | 25 (92.6) |
| No | 2236 (51.0) | 3979 (90.7) | 3999 (91.2) | 4223 (96.3) | 3322 (75.7) | 3663 (83.5) | 3338 (76.1) | 3868 (88.2) |
| **Have you ever had contact with a TB patient?** |  |  |  |  |  |  |  |  |
| Yes | 120 (60.9) | 184 (93.4) | 186 (94.4) | 194 (98.5) | 174 (88.3) | 171 (86.8) | 169 (85.8) | 185 (93.9) |
| No | 1693 (49.6) | 3119 (91.3) | 3120 (91.4) | 3296 (96.5) | 2607 (76.3) | 2841 (83.2) | 2599 (76.1) | 3016 (88.3) |
| No idea | 438 (54.6) | 700 (87.3) | 717 (89.4) | 758 (94.5) | 560 (69.8) | 673 (83.9) | 592 (73.8) | 692 (86.3) |
| **Total** | 2251 (51.0) | 4003 (90.7) | 4023 (91.1) | 4248 (96.2) | 3341 (75.7) | 3685 (83.5) | 3360 (76.1) | 3893 (88.2) |

Notes: Q1: what is TB; Q2: how is TB transmitted; Q3: what are the suspicious symptoms of TB; Q4: if you suspect that you have TB, what should you do; Q5: is TB curable; Q6: while at school, when you develop suspicious symptoms of TB or are diagnosed with TB, what should you do; Q7: what practice is beneficial in preventing the spread of TB; Q8: which lifestyle habit will improve your immunity?
